# Recurrent Watershed Infarction Without Evident Intracranial Arterial Stenosis Due to Antiphospholipid Syndrome: A Case Report

**DOI:** 10.7759/cureus.50201

**Published:** 2023-12-08

**Authors:** Masahiro Mimori, Kenichi Sakuta, Shinji Miyagawa, Hiroshi Yaguchi

**Affiliations:** 1 Neurology, Jikei University, Bunkyo-ku, JPN; 2 Neurology, Kashiwa Hospital, Jikei University School of Medicine, Kashiwa-shi, JPN; 3 Neurology, Jikei University Kashiwa Hospital, Kashiwa-shi, JPN

**Keywords:** juvenile cerebral infarction, juvenile stroke, recurrent stroke, watershed stroke, antiphospholipid syndrome

## Abstract

Antiphospholipid syndrome (APS) is an autoimmune disorder characterized by arterial, venous, or small vessel thromboembolic events. We present here a rare case of APS with repeated multiple cerebral infarctions in the same watershed area without visible arterial stenosis. A 53-year-old woman without a past medical history presented with a headache and numbness of the right fingers. Magnetic resonance imaging (MRI) showed acute ischemic lesions in the left middle cerebral artery (MCA) watershed area. Blood tests revealed positive anticardiolipin (aCL) and aCL beta-2-glycoprotein I antibodies (aCL-β2GPI). Three months later, aCL and aCL-β2GPi antibodies were still positive, and APS was confirmed.^ ^After four months from the index stroke, she was suddenly affected by right arm and leg weakness under a warfarin prescription. Brain MRI showed a recurrence of acute ischemic stroke in the same left MCA watershed area and the right cerebellar hemisphere without visible intracranial artery stenosis in magnetic resonance angiography. The examination of carotid ultrasonography, electrocardiogram monitoring, as well as transthoracic and transesophageal echocardiography revealed no abnormalities, indicating that the recurrent ischemic stroke was due to APS. Single-photon emission-computed tomography captured wide hypoperfusion beyond the infarction area. Thus, the stroke may have been caused by a repeated thromboembolic mechanism. In conclusion, APS should be considered a differential diagnosis in repeated watershed strokes without obvious intracranial arterial stenosis.

## Introduction

Antiphospholipid syndrome (APS) is characterized by an increased arterial and/or venous thrombotic tendency [1]. Stroke accounts for 13.1% of the first clinical manifestations of APS, and its cumulative incidence over 200 months in patients with APS was 19.8% [1]. The mechanisms of cerebral infarction in APS are diverse, mainly caused by embolism [2-4]. We report here a rare case of APS that presented with repeated cerebral infarction in the same watershed area without evident arterial stenosis.

## Case presentation

**Figure 1:**
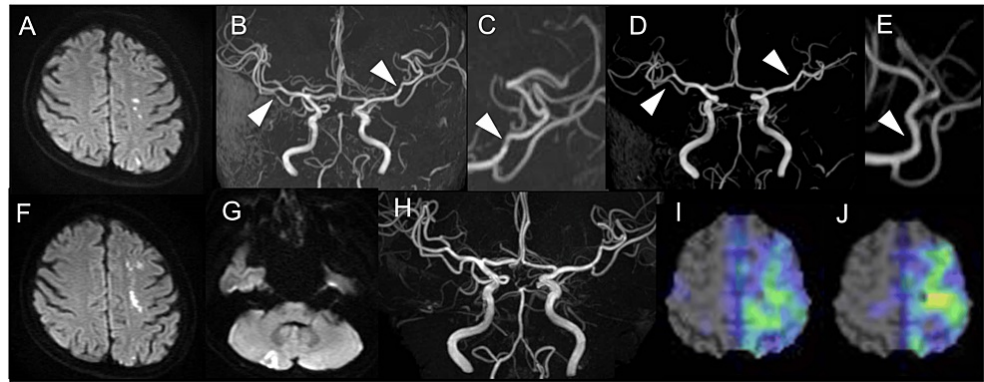
Imaging findings of the two episodes of cerebral infarction and subsequent hospitalization (A) Diffusion-weighted images (DWI) at index stroke showing multiple acute ischemic lesions in the left middle cerebral artery (MCA) watershed area. (B) Magnetic resonance angiography (MRA) at index stroke showing mild stenosis of the bilateral MCA. (C) Magnified image of the left MCA in part B. (D) MRA at nine days after index stroke onset showing improvement of the multiple stenosis. € Magnified image of the left MCA in part D. (F) DWI at the time of the subsequent stroke showing recurrence in the same watershed areas. (G) DWI at the time of the subsequent stroke showing a new infarction in the right cerebellar area. (H) Visible stenosis not evident in MRA at the time of the subsequent stroke. (I) Iodine-123-labeled N-isopropyl-p-iodo-amphetamine Single-photon emission-computed tomography (IMP-SPECT) images at the time of the subsequent stroke showing cerebral blood flow (CBF) reduction in the left MCA area. (J) SPECT images two months after the subsequent stroke. No changes to CBF reduction were observed.

A 53-year-old woman without a past medical history was admitted to our hospital with a headache and numbness of the right fingers. On admission, her neurological examination revealed dysesthesia in her right fingers, disturbed finger motility, and headache in the forehead. Magnetic resonance imaging (MRI) showed acute ischemic lesions in the left middle cerebral artery (MCA) watershed area (Figure 1A) and mild stenosis of the bilateral MCA (Figures 1B, 1C). She immediately started treatment with 200-mg aspirin and 200-mg cilostazol; after 9 days, the magnetic resonance angiography (MRA) showed an improvement in the multiple intracranial artery stenosis (Figures 1D, 1E). A blood test revealed a white blood cell count of 3600/μL, C-reactive protein level of 0.04 mg/dL, PT-INR of 0.94, and positive results for anticardiolipin (aCL) and anticardiolipin beta-2-glycoprotein I antibodies (aCL-β2GPI). These laboratory results and clinical course led to a diagnosis of reversible cerebral vasoconstriction syndrome due to APS. Three months later, aCL and aCL-β2GPi antibodies were repositive, therefore APS was confirmed [5]. Then, she was prescribed warfarin for stroke prevention. Four months after the index stroke, she was suddenly affected by right arm and leg weakness. On admission, her neurological examination revealed moderate weakness in the right extremities and dysesthesia in her right hand, and no obvious ataxia was found. MRI showed acute ischemic lesions in the same left MCA watershed areas and right cerebellar hemisphere (Figures 1F, 1G) without visible intracranial artery stenosis in MRA (Figure 1H). The carotid ultrasonography, electrocardiogram monitoring, and transthoracic and transesophageal echocardiography revealed no abnormalities. Accordingly, the recurrent ischemic stroke caused by APS was confirmed and we increased the dosage of warfarin. Single-photon emission-computed tomography (SPECT) on admission for the second stroke showed reduced cerebral blood flow (CBF) in a wide area beyond the infarction in the left MCA area (Figure 1I). After two months, the SPECT showed no remarkable changes in CBF (Figure 1J). The remaining neurological findings were a mild flexion impairment of the right hand and an abnormal tingling sensation.

## Discussion

The uniqueness of this case is that a recurrent stroke occurred in the same watershed area in a patient with APS without visible arterial stenosis. In particular, the second infarction was a multiple infarction including the right cerebellum, and the PT-INR was not sufficiently prolonged. Therefore, we considered that the MCA watershed infarction at the same location as the first one was caused by an embolism due to APS.

According to the distribution of ischemic lesions, watershed stroke is classified into cortical border zone (CBZ) and internal border zone (IBZ); their corresponding etiology being embolism for CBZ and hemodynamism for IBZ [6]. Small cortical infarcts were observed more frequently in CBZ infarct patients than in IBZ infarct patients [6]. The ischemic lesions, in this case, were located on the CBZ. Common mechanisms of cerebral infarction related to APS are thrombosis [2], embolic occlusion [2], arterial dissection [3], or vasospasms [4]. Previously, cerebral angiography in stroke patients with APS showed multiple intracranial artery stenoses at the peripheral vessels [2,7]. Based on these findings, the decreased blood flow observed in this case may have been due to repeated microembolisms in the peripheral vessels, which are difficult to visualize with computed tomography angiography or MRA.

## Conclusions

APS should be considered a differential diagnosis in cases of recurrent watershed stroke without obvious arterial stenosis. In the present study, we confirmed that CBZ watershed infarcts are also caused by an embolic mechanism. In addition, the area of hypoperfusion was larger than the infarcted foci, and it is necessary to accumulate more cases to confirm whether this phenomenon is specific to the cerebral blood flow of APS patients.

## References

[1] Cervera R, Piette JC, Font J (2002). Antiphospholipid syndrome: clinical and immunologic manifestations and patterns of disease expression in a cohort of 1,000 patients. Arthritis Rheum.

[2] Provenzale JM, Barboriak DP, Allen NB, Ortel TL (1998). Antiphospholipid antibodies: findings at arteriography. Ajnr Am J Neuroradiol.

[3] Iseki T, Yamashita Y, Ueno Y (2021). Cerebral artery dissection secondary to antiphospholipid syndrome: a report of two cases and a literature review. Lupus.

[4] Uenaka T, Hamaguchi H, Sekiguchi K, Kowa H, Kanda F, Toda T (2013). Reversible cerebral vasoconstriction syndrome in a stroke patient with systemic lupus erythematosus and antiphospholipid antibody (Article in Japanese). Rinsho Shinkeigaku.

[5] Miyakis S, Lockshin MD, Atsumi T (2006). International consensus statement on an update of the classification criteria for definite antiphospholipid syndrome (APS). J Thromb Haemost.

[6] Yong SW, Bang OY, Lee PH, Li WY (2006). Internal and cortical border-zone infarction: clinical and diffusion-weighted imaging features. Stroke.

[7] (1990). Clinical and laboratory findings in patients with antiphospholipid antibodies and cerebral ischemia. The Antiphospholipid Antibodies in Stroke Study Group. Stroke.

